# Urticaria of the Hog

**Published:** 1897-05

**Authors:** 


Urticaria of the Hog. Having made a study of this malady, M. Guittard thus gives the symptoms useful in the differential diagnosis of urticaria, rouget, pneumo-enteritis, and anthrax in the hog:

*Urticaria:* The cutaneous spots of a brownish color, not confluent, well-defined, situated upon the back, the neck, the lateral parts of the body, and the rump. A moderately pronounced fever; anorexia; no diarrhœa. Termination of disease usually satisfactory. Attacks young animals especially, and is not very fatal.

*Rouget:* Cutaneous spots of a mottled, violet color, poorly defined and widely extended, on the ears, the neck, beneath the belly, on the flanks, and extending often over one side of the body. A very pronounced fever; evolution very rapid. The diarrhœa is serous and sometimes bloody. Respiration very much accelerated. Complete anorexia. Termination almost always fatal. It attacks only animals of adult age.

*Pneumo-enteritis:* Attack and development gradual; prostration pronounced; flatulency; constipation at the end, then a fetid diarrhœal discharge mingled with blood. Cough, throwing up, with difficulty of respiration. Hemorrhagic and œdematous spots on the ears, the thighs, and beneath the belly. Attacks animals of all ages, and is very fatal.

*Anthrax:* Diffuse congestion under the throat; œdematous, mottled with violet, and increasing rapidly. Œdema on the surface; deglutition difficult; general weakness; diarrhœa developing very rapidly. Asphyxia threatening. Death almost certain. Occurs very rarely.—*Annales de Méd. Vét.*, March, 1896.

